# Delayed pulmonary embolism after unicompartmental knee arthroplasty: A case report

**DOI:** 10.1097/MD.0000000000024230

**Published:** 2021-01-08

**Authors:** Yun Guan, Zhimin Zeng

**Affiliations:** Medical center, Ningbo NO. 6 Hospital, Ningbo, Zhejiang, China.

**Keywords:** arthroplasty, knee, pulmonary embolism, thromboprophylaxis

## Abstract

**Introduction:**

Although venous thromboembolism (VTE) is relatively rare after unicompartmental knee arthroplasty (UKA), symptomatic pulmonary embolism (PE) can be fatal. Whether routine thromboprophylaxis or thrombolytic treatment is necessary for patients undergoing UKA remains unclear. Here, we present a case of delayed pulmonary embolism after UKA.

**Patient concerns:**

A 57-year-old women underwent cemented UKA for left localized medial knee pain. There were no risk factors of VTE besides high BMI before surgery. 2 months after surgery, the patient presented with dyspnea and palpitation, and these symptoms could not be alleviated after rest.

**Diagnosis:**

An arterial blood gas analysis showed decreased PO_2_, SO_2_ and PCO_2_. Pulmonary CTA showed multiple pulmonary embolism in the trunk of the right lower pulmonary artery and the branch of the left lower pulmonary arteries. The final diagnosis was delayed pulmonary embolism after UKA.

**Interventions:**

Urokinase thrombolysis was administered intravenously. Low molecular weight heparin and warfarin were prescribed for anticoagulation.

**Outcomes:**

The patient's symptoms abated, and chest CTA showed that the pulmonary embolism had dissolved. No further thrombosis has been observed for more than 6 years.

**Conclusions:**

We presented an unusual case of delayed pulmonary embolism after UKA. Despite the low incidence, its life-threatening nature makes it imperative for surgeons to be well-informed about thrombosis and pay more attention to its prevention strategies.

## 1 Introduction

Venous thromboembolism, which could manifest as either deep vein thrombosis (DVT) or pulmonary embolism, is one of the most common complications following total knee arthroplasty (TKA). Today, using current VTE prophylaxis, the incidence of postoperative VTE following joint arthroplasty surgery has decreased to 0.28% in Asian patients.^[1]^ However, to the best of our knowledge, there is limited data on the incidence of DVT and PE after unicompartmental knee arthroplasty. Owing to the superior functional results and fast recovery, UKA is becoming an increasingly popular alternative treatment for unicompartmental knee osteoarthritis.^[2]^ Recent studies have reported the prevalence of VTE following UKA in patients receiving routine pharmacological thromboprophylaxis is remarkably lower than that following TKA,^[3,4]^ Koh et al even suggested that routine thromboprophylaxis or thrombolytic treatment in Korean patients undergoing UKA may not be necessary.^[4]^ However, this complication can be regarded as life-threatening; Here, we present a case of delayed PE confirmed by CTA after UKA.

## 2 Case report

**Figure 1:**
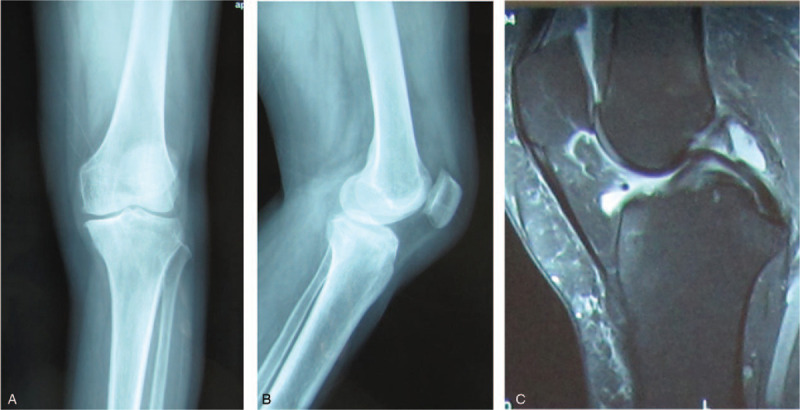
a, b: Anteroposterior and lateral views of the left knee before UKA, showing localized medial unicompartmental osteoarthritis. c:MRI showing intact ACL and PCL.

**Figure 2:**
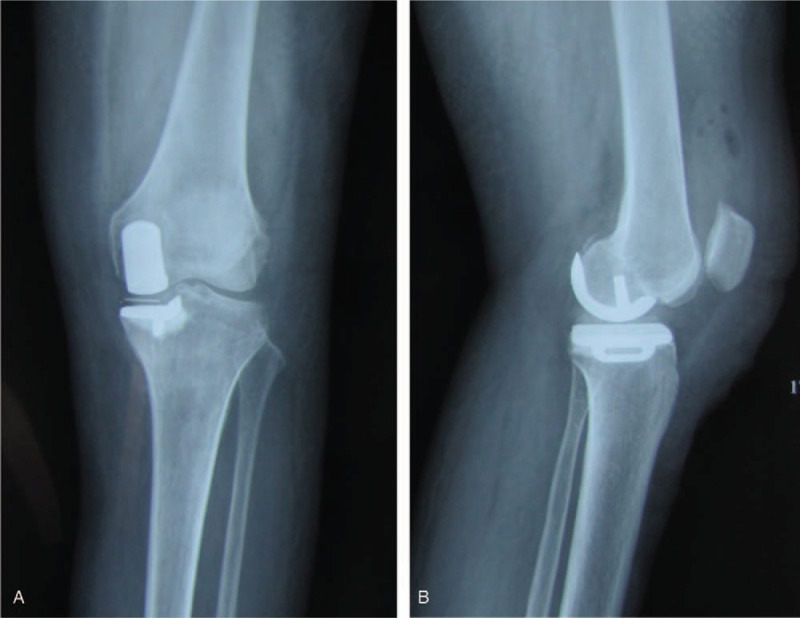
a,b: Anteroposterior and lateral views of the left knee after UKA, showing good implant positioning.

A 57-year-old women underwent cemented UKA for left localized medial knee pain. Preoperative plain radiograph showed medial unicompartmental knee OA and no degenerative change in other compartments (Fig. 1a,b). Magnetic resonance image (MRI) showed continuity of the anterior cruciate ligament (Fig. 1c). She had no history of VTE or vascular diseases, and the BMI was 32.1 kg/m^2^. The operation was done successfully under single-shot spinal anesthesia. Oxford III mobile bearing UKA was employed for this patient. A pneumatic tourniquet was used during surgery. Postoperative plain radiograph showed a well-aligned knee and good implant positioning (Fig. 2a,b). As a routine procedure of thromboprophylaxis, pneumatic compression device was applied immediately after surgery. The patient was encouraged to start her ambulation with full weight bearing on the day of surgery, and received 10 mg rivaroxaban daily for 14 days postoperatively.

**Figure 3:**
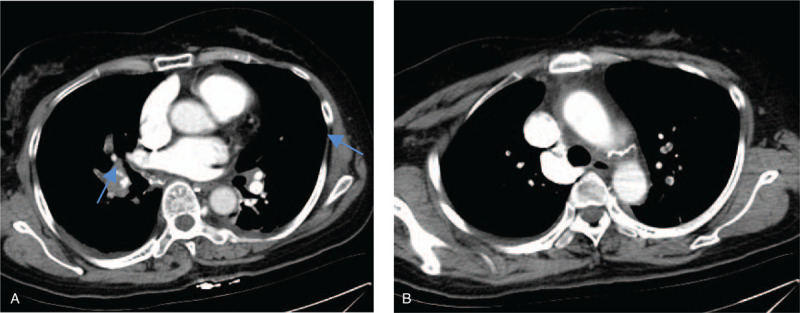
a,b: pulmonary CTA, showing multiple pulmonary embolism in the trunk of the right lower pulmonary artery and the branch of the left lower pulmonary arteries.

Two months after surgery, the patient presented with dyspnea and palpitation, and these symptoms could not be alleviated after rest. An arterial blood gas analysis showed decreased PO_2_ (26 mm Hg; normal, 80–100 mm Hg), SO_2_ (87.2%; normal, 95–100%) and PCO_2_ (25 mm Hg; normal, 35–45 mm Hg). Laboratory data showed D-dimer was 1954 ng/mL (normal, 0–243 ng/mL), and pulmonary CTA showed multiple pulmonary embolism in the trunk of the right lower pulmonary artery and the branch of the left lower pulmonary arteries (Fig. 3a,b). She was admitted to the endovascular department with the diagnosis of pulmonary embolism. Urokinase thrombolysis (200000 units ivgtt in 10 minutes, followed by 1200000 units ivgtt for 10 hours) was administered intravenously. Low molecular weight heparin (0.6 mL subcutaneous injection, every 12 hours) and warfarin (take 4.375 mg once a day in the evening) were prescribed for anticoagulation. One week later, the patient's symptoms abated, and chest CTA showed that the pulmonary embolism had dissolved. At the time of writing this report, she has been alive for 6 years, and no further thrombosis has been observed.

## 3 Discussion

VTE and PE remain a major potential complications after total knee and hip arthroplasty, and its incidence has decreased recently, partly owing to routine mechanical and chemical prophylaxis.^[1]^ But no consensus has been reached about thromboprophylaxis after UKA. UKA is widely used to treat isolated unicompartmental knee OA. Compared to TKA, UKA is less invasive and has a lower complication rate and faster recovery time.^[5]^ Initial reports suggested that the incidence of DVT after UKA was within the range of data reported for TKA, or lower.^[6]^ Recently, the prevalence of VTE after UKA in Korean patients who do not receive thromboprophylaxis was 26%, and all VTEs were clinically asymptomatic and spontaneously regressed within 6 months; Koh et al suggested that routine thromboprophylaxis treatment in Korean patients undergoing UKA may not be necessary.^[4]^ However, the present study reports an unusual case of delayed PE after UKA, as confirmed by CTA.

According to the VTE prophylactic guidelines, old age (≥ 75 years), high BMI (≥ 30 kg/m^2^), history of venous thromboembolism, cerebrovascular disease, history of malignancy and long-term use of oral contraceptives or hormone replacement therapy were risk factors to thrombosis.^[7]^ The patient was 57 years old, she had no history of VTE, malignancy, or use of hormone, and she had no risk factors other than high BMI (32.1 kg/m^2^). Several studies were published concerning the time span between surgery and the thromboembolic event after arthroplasty; White et al stated that postoperative day 7 is the median time for symptomatic DVT after primary TKA, and the risk of asymptomatic DVT could last for up to 3 months after surgery.^[8,9]^ In this case, the patient was encouraged to start her ambulation on the day of surgery, and received 10 mg rivaroxaban daily for 2 weeks. However, she presented with the typical clinical manifestations of PE from postoperative 2 months, which was subsequently confirmed by pulmonary CT. However, she survived with the help of timely thrombolytic and anticoagulant therapy.

A meta-analysis showed that aspirin provided comparable VTE prophylaxis compared with factor Xa inhibitors with the lowest risk of bleeding.^[10]^ This is supported by a prospective study conducted by Schmidt-Braekling et al that demonstrated that aspirin in combination with in-hospital pneumatic compression is an adequate DVT prophylaxis treatment after UKA.^[11]^ Considering that aspirin is relatively safe and affordable, the duration of application after UKA may be extended. In addition, surgeons must be well-informed about VTE and PE to reduce the incidence rate and hazard of this complication.

We presented a rare case of delayed pulmonary embolism after UKA in a 57- year-old woman, who was successfully treated with timely thrombolytic and anticoagulant therapy. Despite the low incidence, its life-threatening nature makes it imperative for surgeons to pay more attention to thrombosis and its prevention methods.

## Author contributions

**Writing – original draft:** Yun Guan.

**Writing – review & editing:** Zhimin Zeng.
